# Essays on Surgery and Midwifery

**Published:** 1822-06-01

**Authors:** 


*Essays on Surgery and Midwifery; with Practical Observations, and Select Cases.* By James Barlow, Surgeon. One Volume, 8vo. pp. 417.

Perhaps as daring and difficult operations in surgery were performed thirty years ago as now; but they are, at present, frequently performed in country towns and even villages, where they would not have been dreamt of half, or even a quarter of, a century ago. Medical and surgical science is therefore rapidly diffusing itself among, what have been termed, the lower orders of the profession, and this, we conceive, is a matter of far greater importance to society at large, than brilliant discoveries in knowledge, when that knowledge is confined in its practical application, to limited circles, or large cities. The provincial press has, of late years, exhibited unquestionable proofs of the respectable state of medicine and surgery in the country, and we are disposed to think, that few surgeons in this proud metropolis would dislike to be considered the author of the work now open before us, though printed and composed in the humble town of Blackburn, in Lancashire. Mr. Barlow appears to be an old, an able, and an experienced practitioner, combining the ardent zeal of youth with the cautious judgment of age—having been long in the habit of attending, not only to the practical rules which experience and observation have supplied, but to the *principles* involved in their application and established by their success. Nothing can be more true, than that "the more the efforts of a practitioner, in surgery, (and he might add, *in medicine*,) are directed by an accurate knowledge of physiology and anatomy (morbid as well as simple,) in their varied and interesting details, the greater will be the certainty of eventual and permanent respectability and success." We have often, as well as Mr. Barlow, had occasion to deplore the too frequent neglect of those pursuits which connect the attainment of skill with the study of science; but, with him also, we rejoice that the diffusion of knowledge, and the enactments of the Legislature, have now secured an increasing attention to every branch of professional information.

The volume before us embraces two very different and distinct subjects—lithotomy and midwifery. It is to the first of these only, that we shall direct our attention in the present article, reserving the latter for our next number.

1. The subject of lithotomy is preceded by some useful preliminary observations on certain disorders of the urinary organs connected with stone in the bladder, which we must not pass over unnoticed. Mr. B. justly remarks, that the anatomy, physiology, and morbid sympathies of the pelvic viscera, should be carefully studied by the operative, and also the medical surgeon. We all know that there is a progressive evolution which marks the range of human existence, and appears to govern the order in which diseases assail the different organs and structures of the body. The prostate gland and neck of the bladder, from their vascular texture and situation, are much exposed to disorder, as life advances. Among the principal causes, we may enumerate high living, constipation of the bowels, and excessive venery. These excite undue vascular action of the parts, and the prostate gland enlarges, partially blocking up, as it were, the orifice of the bladder, so that the urine seldom gets completely evacuated, and a sediment is left behind. Where a predisposition to form calculus exists, portions of gravel, which pass the ureters, are retained in the cavity of this receptacle till a nucleus is formed, and a stone, too large to pass the urethra, may thus be produced. Retention of urine, under such circumstances, is not unusual, and the aid of the catheter becomes necessary. The symptoms of diseased prostate and calculi are so associated, that no diagnostic can be formed without an actual examination, either by the finger passed up the rectum, or by sounding the bladder. In cases where there was much pain or irritation in the bladder from the presence of calculi, or morbid affection of its coats, accompanied with irresistible efforts to micturate, and pain in the glans penis, or neck of the bladder, our author has found much benefit from injections, into the bladder, of solutions of opium and belladonna in mucilage of acacia, diluted with tepid water. We wonder that this measure is not more frequently resorted to, were it only to allay the acrid stimulating property of the urine, which acts so detrimentally on the too irritable lining of the bladder. Sometimes the introduction of a bougie is useful, in lessening this morbid irritability. In not a few instances, the prostate gland is invaded with irregular swelling, increasing the prostatic curve of the urethra, and thus distorting the passage, so as to cause permanent retention of urine. Sometimes, our author has succeeded, in such cases, by employing a catheter less curved than ordinary—on other occasions, more bent, assisted by the finger in the rectum. Active inflammation of the prostate gland is another perplexing cause of retention. In these cases, the tender vessels of the urethra become so surcharged with blood that, on every attempt to introduce the catheter, the eyes of the instrument get blocked up, and no urine can pass.

"To obviate any obstruction in the catheter, I usually plug up the eyes of the instrument with wax before its introduction, and when the point has reached the fundus *vesicæ*, I withdraw the stillet, and if the wax does not dissolve by the heat of the part in due time, I then place my mouth to the open end, and by blowing forcibly through the tube, the wax will be forced out, and the urine pass off without further interruption. In other cases, where the eyes of the catheter have not been previously plugged, and where the urine does not pass voluntarily, I adopt the same expedient, and generally with the same success." 16.

In most cases of diseased prostate, or bladder-affections with retention, our author has been in the habit of keeping the *flexible pewter catheter* in the bladder, "by passing the instrument in the usual way, and then introducing a finger up the rectum, pushing it upwards, so that the concave part, near the point, will hook behind the os pubis, and remain permanently fixed." It should be removed and cleaned, of course, once in six or eight days.

In over-distention of the bladder, and loss of power in its muscular fibres, of some days duration, the catheter becomes an uncertain remedy, as the ureters and kidneys sometimes participate in the state of the bladder, their valvular apparatus becoming changed, and the secretory function suppresed. Mr. Barlow thinks that, if a dribbling of urine did not take place, the bladder would more frequently fall into gangrene than we find to be the case. Early and prompt attention on the part of the practitioner is necessary on such occasions, to prevent vesical and abdominal inflammation. If the bladder ulcerates towards the abdomen, the case is lost—but when the constitutional symptoms are not very grave, and the bladder gives way below that portion of the organ, which is screened by the peritoneum, "the surgeon should immediately proceed to make a dependant opening into the cellular membrane, where the extravasated urine is lodged, and evacuate the fluid; and to promote the healing of the wound of the bladder, a catheter should be kept constantly in the passage of the urethra, lest a fresh supply of urine from the ureters be accumulated in the cavity, and prevent the process of cure."

These cases require strict attention to the antiphlogistic course, besides mechanical aid. Bleeding, local or general, is necessary where there are pyrexial symptoms. The bowels should be emptied by brisk cathartics; and, if there exist much pain and irritation about the neck of the bladder, the tepid bath, and leeches are useful. A combination of the extract of conium, hyoscyamus, colocynth, and blue pill, may be given every night and morning, with effervescing draughts, antimonials, and digitalis, in the day. Our author has experienced great benefit, in such cases, from alternately injecting large portions of warm and cold water up the rectum. But we dare not dwell any longer on the judicious practical observations contained in these preliminaries.

The next subject discussed by Mr. Barlow, is the symptomatology of stone in the bladder, together with the method of sounding. This section, however, we must pass over, though containing much sound remark. Our author then gives a succinct account of the different modes of operating for the stone, beginning with the Celsian, or apparatus minor, and ending with that by the knife alone. On each method, our author makes some remarks of his own. The Celsian operation, or "cutting on the gripe," Mr. Barlow is induced to think, by no means an uneligible operation, when confined to young subjects. "The method is simple, and less burthened with instruments than any other plan of operation by the lateral passage, and greatly resembles the mode adopted by Cheselden and Raw." Celsus appears to have been well aware of the danger attending a small wound, and the retention of urine, which sometimes succeeds an operation.

"Proximo die," says he, "si spiritus difficilius redditur, si urina non excedit, si locus circa pubem mature intumuit, scire licet, in vesica sanguinem concretum remansisse. Igitur, demissis eodum modo digitis, leniter pertractanda vesica est, et discutienda, si qua coierunt: quo sit, ut per vulnus postea procedant." 69.

He recommends the stone to be broken, if too large, a practice invented by Ammonius, about 150 years subsequent to Hippocrates, and thence called lithotomy, or stone-cutting.*

Passing over the apparatus major and those "horrid instruments called dilators," our author comes to the high operation, lately revived in this country. He properly observes, that as this operation has, for a long time, been abandoned, we can only judge of its success, from the records of those times in which it was in repute, compared with the present lateral operation.

"And if I mistake not," says Mr. B. "the success attending the High Operation as practised both by the English and French sur-

---

* "Si quando autem is major non videtur, nisi rupta cervice, extrahi posse, findendus est, cujus repertor Ammonius, ob id λιθοτομος cognominatus est." *Cels. lib.* vii. *p.* 44.

The writings of Celsus may be considered as exhibiting an epitome of the medical and surgical knowledge of the Antients at the time he wrote. We have always been more surprized at the intrepidity of their surgery, than the depth of their physic. Considering that they were ignorant of the circulation of the blood, we cannot but admire their bravery in extirpating the thyroid gland—at least, Celsus describes the removal of bronchocele by the knife.

"At in cervice, inter cutem et asperam arteriam, tumor increscit (bronchocele Græci vocant) quo, modo caro hebes, modo humor aliquis, melle aquæve similis, includitum. ***Potest autem adurentibus medicamentis curari. **Sed scalpelli curatio brevior est. Medio tumore una linea inciditur usque ad tunicam: deinde vitiosus sinus ab integro corpore digito separatur, totusque cum velamento suo eximitur." *Lib.* vii. *Sec.* 13.

It is very remarkable, that the Antients should have been in the habit of using the ligature to vessels that were divided in *wounds*, and yet that they, and we may say the Moderns, till comparatively of late, should never have thought of tying the vessels in amputation. That the ligature was familiar to Celsus, is proved by the following passage. After describing various styptics for stopping hæmorrhage from wounds, he goes on to say:—

"Quod si illa quoque profluvio vincuntur, venæ (by which he means all blood-vessels) quæ sanguinem fundunt apprehendendæ, circaque id, quod ictum est, *duobus locis deligandæ, intercidendæque sunt*, ut et in se ipsæ cöeant, et nihilo-minus ora preclusa habeant."—*Lib.* v. *de Vulneribus.* Here is the modern operation of tying the artery in two places, and dividing it between the ligatures, in the most clear and unequivocal language. Both the above extracts shew, that Mr. S. Cooper is not justified in saying, (art. Hæmorrhage,) that "the Ancients, ignorant how to stop bleeding, were afraid to cut out the most trivial tumour."—*Dict.* *3d Ed. p.* 518.

geons at that period equalled, if not surpassed those of the same hospitals, in both countries at the present day, when performed under similar circumstances." 87.

Although our author thinks that it becomes a question whether the high or the lateral operation is to be preferred, yet he properly remarks that neither of them should be indiscriminately and exclusively adhered to—"for each has its advantages of being peculiarly adapted to a distinct mode of operating, according to circumstances." We have seen both operations performed, and we have conversed with several of the first surgeons in this metropolis respecting the high operation; but they all seem to agree that it will never come into general use, but should be limited to those cases where the diseased state of the prostate gland, or the magnitude of the calculus presents insuperable objections to the lateral mode of extraction.

Mr. Barlow appears to be sceptical as to the encystment of stones in the bladder. No such event has occurred to him. He is induced to think that the incidental embarrassments sometimes attending the operation of lithotomy are too frequently imputed to such morbid occurrences, as an apology for inadvertence or unskilful practice. It is certain that many authors have doubted the existence of sacculation —among others, Rousset, Collet, and Tolet; but the circumstance has been attested by too many qualified and credible witnesses to be now disbelieved. Stones, we know, are liable to lodge in the angle behind the prostate gland, and there they may become encysted. Mr. Barlow has furnished us with a plate of a lever or scoop, which appears to be well calculated for exploring the cavity of the bladder, and detaching sacculated stones.

Our author gives a concise history, and description of the lateral operation, to which we refer our surgical readers. At page 126-7, we have the following important remarks:—

"The chief object to be attained in lithotomy, by whatever form of instrument it be accomplished, is to make the incision from its commencement in the integuments below the scrotum to its termination below the anus, and through the intervening muscles, prostate gland, and neck of the bladder, to an extent proportioned to the size of the patient, and sufficiently to admit the forceps, and extraction of the stone without bruising or lacerating the contiguous parts. But this cannot be effected unless the whole of the incision be made more extensively than what most modern operators are in the habit of doing, and hence, I am persuaded, results the disparity of success.

"An ample division of the parts concerned in the operation always facilitates the extraction of the stone, discharge of urine, or blood, from the wound, prevents subsequent inflammation, and greatly expedites the patient's recovery.

"On comparing the cutting part of most gorgets now in use, with the extent of the prostate gland, from its apex to the base, where it surrounds the cervix vesicæ, it will be evident that such construction is inadequate to effect a complete section of this organ even in its natural state; but when enlarged by disease, as it frequently is in calculous affections, such defect is still more manifest and the danger resulting is proportionably greater. Under such circumstances is it possible, without incurring danger to the patient, to extract a stone from the bladder, the dimensions of which, together with the blades of the forceps far exceed that of the wound through which it should pass with freedom? I have more than once seen great violence used in extracting the stone through an inadequate incision, and was present at an operation in St. Bartholomew's Hospital in London, where the operator used such force in extracting the stone, that he would have fallen backwards had he not been supported at the time by his assistants. It is easy to foretel the fate of a patient so unfortunately circumstanced.

These reflections are intended to lead surgeons to pause on the propriety of using the gorget, and to induce them to select an instrument, (let its name be what it may,) which will invariably make a section of the prostate gland sufficiently extensive for the free extraction of the stone, without going beyond the limits of safety; for it should always be remembered, that the edge of the gorget can never be made to cut the substance of the gland freely, owing in part to its receding by the impulse of the instrument, and leading the surgeon to suppose that he has completed a section of this organ, when in reality he has merely divided its apex." 128.

It is perhaps to be lamented, as our author observes, that human ingenuity should have been so much exercised in the construction of an instrument (the gorget) that is designed to substitute mechanism for anatomical knowledge, and the skill of the cutler for the dexterity of the surgeon. We know, indeed, that in the hand of a COOPER, any instrument will do the business well—but Heaven defend us from the dark and direful plunge of the gorget, when directed by any but the hand of a master! Mr. Barlow considers that, when the bulk of the stone equals that of a hen's egg, the wound by the gorget must be clearly inadequate to admit the stone and mouth of the forceps, without lacerating the cervix vesicæ, or wounding the adjacent parts. These accidents are known to have occurred in the hands of eminent surgeons.

The instrument which Mr. Barlow has mostly used in lithotomy, is the Bistouri Caché of Frere Come, with an additional beak, to prevent its injuring the bladder. He has operated on upwards of *sixty* patients for the stone with this instrument, (and one hereafter to be mentioned as a substitute for the gorget,) out of which number *two* only died. This we consider as very great success indeed.

"Objections have been advanced by writers regarding the simplicity and form of the bistouri, and manner of dividing the parts concerned in the operation. Truth, however, urges me to state, that I have neither found difficulty nor danger attending its application; as the success I have experienced will best decide. I have operated with it both on men and women, from little more than two years old, to upwards of seventy, and extracted stones more than five ounces in weight. It will not then, I trust, be hereafter said, that the bistouri caché is an instrument in any respect less eligible or safe than either the gorget or knife, when used with judgment." P. 131.

Our author has given a plate of a beaked lithotomy knife or bistoury, which appears to him to combine in itself all the advantages of the *gorget*, *knife*, and *probe-pointed bistoury*, without their imperfection. He has operated with this instrument several times both on old and young subjects with perfect ease and success; and from its simplicity and manner of dividing the necessary parts, he has no doubt that it will hereafter supersede every other instrument yet invented. We must refer to the plate for a delineation of this knife.

*After-Treatment.* This is of the utmost consequence. Hæmorrhage and inflammation are the two principal objects of our attention. The state of the bladder should be frequently inspected, and care taken that it does not become distended. A low diet, cool apartment, and daily injection, should be prescribed. Should restlessness, anxiety, quick pulse, thirst, pain, and tension in the pubic region occur, every means of subduing inflammation should be pursued. Our author properly remarks that a feeble pulse is a frequent attendant on all abdominal inflammations—the practitioner ought not therefore to be deterred from the use of the lancet, by such fallacious symptoms.*

*Hæmorrhage* has never occurred in our author's practice, though he often hears of it in the practice of others. He thinks it can only happen from unskilfulness or some unusual distribution of the blood-vessels. We shall here pre-

---

* Depletion, indeed, in such cases, ought to be persevered in while life remains, or the bad symptoms continue. We were lately in attendance with Sir Astley Cooper, where pelvic and peritoneal inflammation continued long and obstinate—indeed, till there were strong indications of abdominal effusion—yet we used the lancet when the pulse was devoid of all strength and hardness, and when the gentleman was blanched by previous venesections. Success ultimately crowned our efforts. The clear judgment of Sir A. Cooper was as evident in this dilemma, as his dexterity in the operation.

sent our readers with a sketch of a case related by our author, which was attended with some unusual symptoms, and has called forth several practical remarks.

*Case.* In the year 1806 Mr. Barlow was consulted by a stout, corpulent, robust man, then about 60 years of age, in consequence of great pain in passing urine, and propulsive efforts to expel it. For some years previously he had occasionally discharged both blood and mucus from the urethra. Sounding was proposed, but rejected; and the patient did not again present himself till 1811, when Mr. B. found him with a two days retention of urine, considerable pain in the region of the bladder with tension, and a dark livid colour of the scrotum. The catheter was introduced, and its point struck against a stone. Nearly two quarts of dark coloured urine were drawn off, and afforded temporary relief. The warm bath, laxative glysters, and gentle aperients considerably mitigated the soreness in the hypogastric region—still the retention of urine continued, caused, as was imagined, by the presence of a calculus lodged in the vicinity of the neck of the bladder. The urine was drawn off twice a day during several successive days. A flexible metallic catheter was now introduced, and hooked upon the pubis, with a cork fitted to the aperture, so that the patient could evacuate his water *ad libitum*.

The prostate gland was found to be greatly enlarged, and in a very rigid condition.* Notwithstanding strict antiphlogistic measures, there remained much soreness in the pubic region, with quick pulse and fever, and therefore lithotomy was not proposed. But these symptoms gradually abated, and the operation was deemed admissible.† On the 17th November the stone was extracted, in the pre-

---

" * The presence of a stone in the bladder may generally, by attention to the symptoms, be distinguished from an irritable or indurated state of that viscus; for in the former affection, and during the discharge of urine, the pain increases at the glans penis till the last drop is voided and subsides gradually; while in the latter case, relief is observed to take place in a reverse ratio, for as soon as the water begins to flow the patient is somewhat relieved, and when the whole is evacuated, there is comparatively little pain left, and the expansive functions of the bladder are sooner restored." 143.

† Our author observes that simple irritation excited by calculi is not an insuperable objection to lithotomy, *when performed during an interval of pain.* The existence, however, of nephritic affection, or insidious morbid action lurking in any organ of importance, increases the danger of the operation, which should be delayed, if possible, till such symptoms are removed, and the irritation subside.

sence of two assisting surgeons. As the operation was one of no common difficulty, we shall give it in the words of the author himself.

"The first stages of the operation were conducted in the usual manner, and with tolerable facility. On the membranous portion of the urethra being laid open with the scalpel to the commencement of the prostate gland, the beak of the bistouri caché was inserted into the groove of the staff, the handle of which was taken hold of with the left hand, and raised from the right groin of the patient to nearly a right angle with the body; the bistouri was then pushed gently forwards into the bladder and the staff taken out; the cutting edge being raised from its sheath and turned rather laterally towards the left ischia of the patient, it was withdrawn nearly in a horizontal direction; and in executing this step of the operation, I perceived an unusual resistance and grating, as if cutting through a cartilaginous substance.

"The fore finger of the left hand was now passed as high as possible into the bladder, through the opening made by the bistouri, and with difficulty the surface of a stone was felt; for owing to the patient's state of corpulency, the chief part of the hand became buried in the wound.

"The forceps were then carefully introduced by the side of the fingers, which served as a guide to seize the stone in the bladder. The fingers being withdrawn, the stone was taken hold of by the blades; but from the great expansion of the handles, I was led to believe that the calculus was either very large, or otherwise seized in an unfavourable direction. To ascertain the fact, I endeavoured to reach the stone, by insinuating the finger betwixt the extended blades of the forceps, but was prevented by the bulk of the prostate gland; for it appeared to occupy so considerable a space, that its extent could not be wholly traced by the finger in any direction. I therefore judged it expedient to quit the stone, and attempt to seize it in a less diameter, and after using every possible means in my power, I was obliged to abandon this project, and the extent and rigidity of the prostate, and its unyielding structure, induced me to enlarge the incision. On every attempt to extract the stone, the body of the gland was brought forwards into sight, and appeared to completely wedge up the space betwixt the two *rami ischii*. Thus situated, and while the left hand was employed in drawing forwards the forceps along with the stone, the right was engaged in dilating* the wound with the scalpel, in a line with the external in-

"* Though in this instance, the incision made by the bistouri caché was inadequate to effect a complete division of the prostate gland, to afford the stone a free exit: yet its extent was far greater than what could be made with the gorget; hence may be seen the advantage which the bistouri possesses over the gorget; for the surgeon has it in his power to adapt the blade of the bistouri to the exigency of every individual case, prior to its introduction into the bladder, and should the cision, where the resistance opposed the greatest obstacle; in this manner sufficient room was made, and the transmission of the stone effected. It was of an oval shape, and its long diameter 2.25, and its short 1.75 inches. A female sound was then passed into the bladder, and another stone detected larger than the first, and which was extracted with proportionate difficulty. It was also oval, but measured 2.6 inches one way, and 2.1 the other. From the different situations in which I had an opportunity of recognizing the prostate gland of this patient, both by the finger passed up the rectum, and through the wound in perineo, its lateral lobes evidently pressed considerably on the rectum, and appeared the shape and size of the gizzard of a goose. Several arteries were divided in the operation, which required the ligature, and there remained a considerable oozing of blood, which appeared to come from the divided edges of the prostate gland: to suppress which a canula, with a piece of sponge wrapped round it, was introduced into the wound, and by its pressure on the incised portions of the gland, prevented the blood from making its way into the bladder, and soon stopped the bleeding." P. 147.

On being put to bed, sixty drops of tincture of opium were given him; but it did not procure sleep. He appeared restless in the evening, with quick pulse; but there was no tension, or hæmorrhage, the water passing *guttatim* through the canula. He was put into the warm bath for twenty-five minutes, which afforded temporary relief, but did not reduce the pulse. The opiate was repeated—no sleep through the night. Aperients, glysters, and the warm bath were exhibited every day for several successive days.

"On the 20th instant, three days after the operation, a degree of soreness and tension manifested itself in the lower part of the abdomen, which extended along the urethra, and assumed the appearance of peritoneal inflammation. But on a minute investigation, I was convinced that the tension of the abdomen was caused by the parts of the wound connected with the operation being distended with inflammation, which wholly prevented the evacuation of the bladder, and the voluntary power of the abdominal muscles from propelling the urine through the aperture.* Without hesitation, I

---

stone be found either of unusual magnitude, or less than what was suspected prior to commencing the operation, the screw of the handle may be elevated or depressed whilst the apex is in the bladder, and the cutting edge regulated to any given extent."

" * I am induced to believe, that *Ischuria Vesicalis*, subsequent to the operation of lithotomy, is not a very rare occurrence, but is frequently the primary cause of Peritoneal Inflammation, though scarcely noticed by authors who have written on the after-treatment of patients. As the symptoms at first view bear much analogy to each other, it is incumbent on the surgeon, in every instance of abdominal affection to passed a female catheter up the wound in perineo into the cavity of the bladder, and evacuated more than a quart of limpid urine, of healthy appearance. This mode of assisting nature in relieving herself, was found necessary to be repeated every eight or ten hours for several succeeding days, until the tension and inflammation of the parts connected with the wound had subsided; after which the urine passed through the artificial aperture with comparative freedom." 149.

In about three weeks from the date of the operation a little urine came away at intervals by the urethra, and the man seemed to be recovering, when a new train of symptoms came on, accompanied with inflammation and suppuration of the right testicle, which produced some fever and irritation in the system; but this was got over; and in ten weeks the patient returned home apparently in good health—the size of the prostate gland, as examined, *per rectum*, much diminished. It is probable, our author thinks, that, had the gorget been used in this case, it would not have divided more than half of the left portion of the prostate gland, and that consequently the incision must have been quite inadequate to the free extraction of the calculus, and probably too small to admit the blades of the forceps without much violence or even laceration.

For the curious case of lithotomy in a female, complicated with procedentia uteri, procedentia vesicæ, and inversio vaginæ, we must refer to the volume itself. The operations and management do great credit to the surgeon.

The surgical half of this volume concludes with a case of tumour on the nose, introduced by some ingenious physiological remarks on the analogy between animals and vegetables, as well in regard to their structure and functions, as their diseases. There can be no doubt, indeed, that one common principle of vitality pervades the whole of the animal, vegetable, and perhaps we should not be wrong in including, the *mineral* kingdom. It is extremely difficult to say where life ceases to exist in any thing we see in air, on earth, or in the ocean. But we will not bewilder ourselves

---

investigate the state of the bladder, and witness the discharge of urine, either from the wound, or by the urethra. By this mode of enquiry, the morbid retention of urine may be distinguished from abdominal inflammation, and if no urime has passed by either of these apertures, a female catheter should be introduced through the wound into the bladder and the water evacuated, by which means the symptoms will subside, and by omitting this mode of relief, inflammation of the abdominal viscera and bladder would inevitably follow, and there is reason to believe, that serious effects have sometimes ensued from this species of retention of urine being overlooked."

in speculations on those subjects which are apparently, or rather *really*, beyond our reach, while so much remains to be learnt that is possible to be accomplished, and useful when acquired. Life is so short, and so great a portion of that life taken up with the necessary functions for which man is destined in this world, that we deem it imprudent, particularly for medical men, to dedicate more than a very limited portion of time to other than the main objects of their professional researches.*

The subject of the tumour in question, was a respectable schoolmaster of Blackburn, who consulted our author "respecting a morbid rubecund tumour seated on his nose," which had been increasing for thirteen years past.

> "The tumour extended from the superior part of the nose over the *alæ nasi* and apex on both sides down to the lower part of the upper lip to such an extent, that the nostrils and mouth were nearly closed, and when laid down to sleep his breathing was greatly obstructed, unless the tumour was supported by a folding of the pillow placed under it; and when attempting to drink, it became in part immersed in the liquid, unless it was raised by the hand.
>
> "At the lower part of the tumour, rather parallel with the septum narium, the cuticular surface of one projecting tubercle was excoriated, from which there was an offensive exudation." 171.

Our author proposed a total eradication of this disgusting excrescence by the knife. Keeping the natural figure of the nose in his mind, he began the dissection at the superior part of the tumour, as high as the dorsal arch, continuing the incision along the alæ down to the apex on both sides, close to the periosteum and perichondrium, steadily pursuing this project till the whole of the morbid mass was eradicated. The operation did not occupy much time, nor did the patient complain of any pain during the removal of the tumour. The wound was allowed to bleed without interruption, and the hæmorrhage ceased in a few minutes, except from one artery, which required a ligature. The whole surface of the sore was healed in three weeks, and no trace of of cicatrix could be observed, unless by a very near inspection. On dissection, this anomalous tumour exhibited a vascular appearance, and assumed a deep red and livid hue, its structure not corresponding in appearance with any distinct species of tumour, which has been described by authors. Mr. Barlow has given two plates, one representing the pa-

---

* We do not mean *the search after patients*, which some of our brethren, we are sorry to say, consider the "main objects" of their pursuits.

tient before, and the other after, the operation. The contrast is most striking.

Our readers will have seen, by this time, that the portion of the volume which we have analyzed does credit to provincial surgery. In our next we shall bring forward some interesting obstetrical information.

